# Social and environmental determinants of disease uncertainty in obstructive sleep apnea: a dyadic qualitative study on patients and co-residents

**DOI:** 10.3389/fneur.2025.1582173

**Published:** 2025-08-12

**Authors:** Yuqi Niu, Yefan Shao, Linlin Chen, Yali Wang, Shanwen Sun, Xiaochun Zhang

**Affiliations:** Department of Respiratory and Critical Care Medicine, The First Affiliated Hospital of China Medical University, Shenyang, China

**Keywords:** obstructive sleep apnea (OSA), disease uncertainty, co-residents, KJ method, illness uncertainty theory, family systems perspective, qualitative research

## Abstract

**Background:**

Obstructive sleep apnea (OSA) is a prevalent sleep disorder characterized by upper airway obstruction during sleep, leading to significant health issues and reduced quality of life. Despite its increasing prevalence, particularly among middle-aged and older adults, low awareness and treatment rates contribute to a substantial burden of disease uncertainty for both patients and their co-residents. This study aims to investigate the social and environmental determinants of disease uncertainty experienced by OSA patients and their co-residents, focusing on the impact of these factors on health behaviors and access to care.

**Methods:**

The study employed the theoretical model of disease uncertainty as a guiding framework and utilized the KJ method for data analysis. Using purposive sampling, 13 OSA patients and their 13 co-residents were selected to form dyads. Ethical approval was obtained, and informed consent was secured from all participants prior to the study.

**Results:**

The analysis of the interview data yielded seven major themes and 19 sub-themes. (1) “The Shadow of Knowing Little”; (2) “The Fog of Night and Day”; (3) “Symbiotic Suffering”; (4) “The Hidden Costs”; (5) “Delay in Seeking Medical Care”; (6) “Complex Choices”; (7) “Vacancies Calling for Attention.”

**Conclusion:**

The findings underscore that OSA patients and their co-residents face considerable uncertainty related to disease awareness, symptom experiences, medical decision-making, treatment plans, and social support. This uncertainty leads to delays in seeking care and poor treatment adherence. To mitigate these issues, it is recommended to enhance public health education on OSA, improve disease awareness and self-management skills among patients and their families, and better integrate medical resources and social support networks. These interventions should address the social and environmental determinants of health to reduce the burden of disease uncertainty and improve overall health outcomes.

## 1 Background

Obstructive sleep apnea (OSA), characterized by recurrent upper airway obstruction during sleep leading to apnea or hypoventilation, represents a significant global public health concern with rising prevalence (1, 2). In China, approximately 11% of adults are affected by OSA (3), causing excessive daytime sleepiness, impaired quality of life, and increased traffic accident risk (4). Furthermore, OSA is strongly associated with elevated risks of hypertension, type 2 diabetes, cardiovascular diseases, and mortality (5–7). However, persistently low awareness and treatment rates mean many patients remain undiagnosed and experience delayed care (8), which is closely tied to a sense of uncertainty about the disease.

Disease uncertainty, defined as an individual’s inability to effectively identify and comprehend disease-related information and circumstances (9), remains a significant challenge for individuals with OSA and their co-residents. This manifests in varied perceptions of symptoms, severity, prognosis, and treatment (5, 10). Patients often struggle to connect their daytime symptoms with nocturnal apneic events (5), delaying medical attention. At the same time, co-residents, who experience sleep disruption and stress from witnessing apneic episodes, face heightened uncertainty. Crucially, disease uncertainty is linked to mood disturbances such as anxiety and depression in OSA patients (11, 12), which further impacts familial well-being. From a family systems perspective, both patients and co-residents experience a dual burden of uncertainty and negative emotions (13). This burden diminishes quality of life, reduces motivation to seek support or information, and contributes to treatment delays or disruptions (14), ultimately affecting prognosis and recovery. Additionally, uncertainty fosters passive coping strategies (15), exacerbating the disease burden and creating a vicious cycle within the family unit.

Therefore, this study focuses on two central research objectives: To examine how disease uncertainty manifests in OSA patients and their co-residents, and to analyze its impact on their individual and shared experiences within the family system. To identify the key social, behavioral, and environmental determinants influencing disease uncertainty, and to explore how these factors shape coping strategies and health outcomes.

The theoretical model of illness uncertainty, grounded in Lazarus’s stress coping theory (16), serves as the guiding framework for this study. To analyze the complex experiences of OSA patients and their co-residents, the KJ method (affinity diagram method) was employed. Developed by Dr. Kawakita Jirou, the KJ method has been widely adopted in Japanese business and quality control contexts. Unlike methods such as Colaizzi’s, which emphasize extracting essential meanings from lived experiences, the KJ method excels at intuitively synthesizing large volumes of complex, unstructured data. By grouping ideas based on natural affinities, it efficiently clarifies connections and reveals patterns (17). Given the study’s objective to map the multifaceted determinants of disease uncertainty within the patient-co-resident dyad and elucidate their interrelated responses, the KJ method provided a distinct advantage. Its capacity for intuitive synthesis enabled the generation of holistic findings, making it particularly suitable for this research. Accordingly, the study employed the theoretical model of disease uncertainty and the KJ method to organize data. Binary joint interviews were conducted to explore, in depth, the experiences of disease uncertainty among OSA patients and their co-residents. The ultimate intention was to provide valuable references for improving the diagnosis and management of OSA.

This study makes several significant contributions to the field: It deepens understanding of disease uncertainty by exploring its manifestations in OSA patients and their co-residents, focusing on the interplay of social determinants of health, behavioral patterns, and environmental influences. It introduces a dyadic perspective by employing joint interviews to capture the shared and individual experiences of patients and co-residents, offering a holistic understanding of the dual burden of disease uncertainty. It provides actionable recommendations for public health policymakers and practitioners to address disease uncertainty, improve awareness, and enhance the diagnosis and management of OSA.

## 2 Methods

### 2.1 Research design

A descriptive phenomenological study was conducted with the objective of describing and exploring disease uncertainty in patients with obstructive sleep apnea and their co-residents. The study was conducted in accordance with the Comprehensive Standardized Guidelines for Reporting Qualitative Research (COREQ) (18).

### 2.2 Participants

In this study, purposive sampling was employed, with participants (patient-co-resident dyads) being recruited between October 2024 and December 2024 from the Sleep Monitoring Unit of the Department of Respiratory and Critical Care Medicine at a tertiary care hospital in Shenyang, China. The study was granted ethical approval by the Research Ethics Committee of the First Affiliated Hospital of China Medical University Approval No. [2024]1020 and was conducted in accordance with the principles set forth in the Declaration of Helsinki. All participants were informed that their participation in the study was entirely voluntary and that they could withdraw at any time. Prior to the interviews, written and verbal consent was obtained from all participants. The criteria for patients with obstructive sleep apnea and their co-residents at the nadir are presented in Table 1.

**Table 1:** Inclusion and exclusion criteria for patients with obstructive sleep apnea and their co-residents.

Inclusion/Exclusion criteria	Obstructive sleep apnea patients	Co-residents with obstructive sleep apnea patients
Inclusion Criteria	①Age ≥ 18 years old; ②has received more than 7 consecutive hours of PSG monitoring at night and confirmed the diagnosis of OSA (44); ③have basic reading, writing and communication skills; ④Informed consent and voluntary participation in this study.	①Persons who live with the patient and play a major role in the management of the patient’s disease, such as the patient’s spouse, parents, children, and only 1 person is selected for each case; ②Age ≥ 18 years; ③Clear consciousness without cognitive dysfunction; ④Possess normal understanding and expression ability; ⑤Informed consent and voluntary participation in this study.
Exclusion criteria	①Severe infections or diseases of the heart, liver, or kidneys; ②Presence of a history of mental illness, serious cognitive impairment or impaired consciousness; ③Taking medication that affects sleep structure (e.g., antihistamines, antidepressants, hypnotics).	①Suffering from a serious physical illness that prevents you from participating in the research; ②Have a previous history of mental illness.

It is important to note that only patients and their co-residents who met both of the aforementioned NAE criteria were included in the study. The sample size of study participants was determined by data saturation, and the sample size was maintained until the respondent’s information was repeated and no further new themes emerged from the data analysis.

### 2.3 Data collection

The data were collected via semi-structured interviews. The research team comprised nine members, including one senior-ranking expert engaged in the field of respiratory and critical care, five master’s degree students in nursing, one technician in the sleep monitoring room, and two experts familiar with the KJ method. In order to achieve the research objectives, the research team conducted a review of the relevant domestic and international literature, developed a preliminary interview outline based on the theoretical model of disease uncertainty, and made adjustments based on the input of the experts. Pairs of study participants (patients and co-residents) were selected for pilot interviews, and the preliminary interview outline was subsequently modified based on the results obtained. Following this, the formal interview outline was formulated after consulting with the experts (Table 2).

**Table 2:** Outline of interviews with patients with obstructive sleep apnea and their co-residents.

Patients (P)	Co-residents (C)
1. How did you feel when your doctor told you that you had obstructive sleep apnea?	1. How did you learn that the patient had obstructive sleep apnea? How did you feel at the time?
2. How much do you know (understand) about this disease now? Do you feel that there are some things that you do not understand, or some questions that are not clear to you now?	2. How much do you know about OSA? How well do you think the patient understands OSA? What aspects do you think are still unclear or misunderstood by patients?
3. What factors do you think affect your understanding of the disease?	3. What factors do you think influence your understanding of the patient’s disease?
4. What changes have you and your family encountered in your daily life because of the disease?	4. How has the patient’s OSA changed your daily life? How have these changes affected you and your family?
5. how do you personally and your family deal with these uncertainties?	5. How do you cope with the uncertainty that comes with the patient’s OSA? How did you communicate and coordinate with the patient?
6. what would you most like to know about this disease? What do you want to know most about the disease?	6. What would you most like to know about this disease? What knowledge or information about OSA would you most like to have?
7. what aspects of help do you expect most from healthcare professionals?	7. What do you expect most from your healthcare provider?

In this study, face-to-face semi-structured in-depth interviews were employed, with the interviewer (YQ N) familiarizing themselves with the pertinent information in advance to guarantee that the interviews were conducted in an optimal manner. All interviews were conducted in a quiet, distraction-free room to ensure that the interviewees (patient-co-residents) were focused and able to express themselves frankly. Prior to the interview, the researcher provided the respondents with an explanation of the purpose, content and significance of the study. They were also informed of the principles of voluntariness and confidentiality, and a signed informed consent form was obtained. The interviews were conducted using open-ended questions to encourage respondents to express themselves freely. Audio recordings and note-taking were employed to ensure the accuracy and completeness of data collection. To prevent the omission of pertinent information, the researcher would elucidate the queries to the respondents (patient-co-residents) in a timely manner during the interview. The duration of each interview was restricted to 30–40 min to guarantee the respondents’ comfort and the efficiency of the interview process.

### 2.4 Data analysis

The recordings were transcribed in their entirety and cross-checked by two trained researchers within 24 h of the interview. The joint interview analysis was designed to capture and explore the evolving dynamics and relationships between patients and their co-residents, while maintaining the integrity of their individual perspectives. This approach serves to enhance the credibility of the analyses and provides a more profound understanding by delving into the interactions between individuals (19, 20).

The following is a description of the process undertaken for the analysis of the data: (1) Independent analysis and in-depth understanding: Two researchers undertook an independent analysis of the data, listening to the audio recordings on multiple occasions to ensure a comprehensive understanding of the statements made by the interviewees. (2) The data was then subjected to a process of coding and the preservation of context. The statements of significance provided by the interviewees were selected and coded with brief phrases or sentences, with the objective of distilling the key information. (3) Card recording: The original descriptions were recorded in parentheses for the purposes of reference. Concurrently, the initial descriptions were recorded in parentheses to ensure the retention of contextual information. (4) Card recording: The coded content was recorded individually on small, bespoke cards measuring 3 cm by 4 cm. (5) Theme refinement: The affinity diagram method (KJ method) was employed to organize and categorize the coded content, which was then categorized into sub-themes, medium-themes and big-themes through repeated reading and brainstorming. The precise methodology is as follows: select a tranquil setting and utilize a sizable table to disseminate the diminutive cards. Group members should peruse all the cards meticulously on at least three to four occasions to gain profound comprehension of the significance of each code. The next step is to brainstorm and filter out any semantic duplicates, before grouping the remaining cards according to their proximity to each other. The next step is to categorize the themes for each group. These should be written on colored cards and placed in the appropriate group. Subsequently, analogous group themes were categorized as medium themes and indicated with distinct colored cards. Subsequently, the analogous medium-group themes were consolidated into comprehensive themes. (6) Drawing KJ diagrams: the categorized cards were examined from a macro perspective and organized into a visually and logically coherent layout. The KJ diagrams were initially created manually and subsequently refined using computer-aided design software. The organization of themes at each level, along with the descriptions provided by respondents, should be considered. Subsequently, the respondents themselves should be consulted to ascertain the accuracy of the findings.

### 2.5 Quality control

The group’s experts have been engaged in clinical work in the field of respiratory and critical care for a considerable number of years. They possess extensive clinical experience and research experience, as well as proficiency in the utilization of the KJ method. The postgraduate students on the panel had undertaken a systematic study of qualitative research as part of their master’s degree programs, and demonstrated proficiency in communication and data collation. The interviewees exhibited a range of fundamental characteristics and were adequately represented. The data obtained from the interviews were subsequently provided to the respective interviewees for verification purposes, thus ensuring the veracity and precision of the data transcription.

## 3 Results

A total of 13 pairs (patients-co-residents) participated in this study. The mean age of the patients was 44.9 years, ranging from 19 to 64 years (Table 3). The mean age of the co-residents was 45.5 years, ranging from 24 to 62 years (Table 4). Interviews lasted between 30 and 65 min (mean 40 min).

**Table 3:** Characteristics of patients with obstructive sleep apnea (*n* = 13).

Participant	Sex	Age	Educational level	Marital status	Occupation	Place of residence	AHI	Minimum blood oxygen
P1	Male	58	Junior High School	married	Unemployed	Rural	66.1	37
P2	Male	37	Undergraduate	married	Technician	City	93.7	72
P3	Female	26	college	Unmarried	Employee	City	5.9	93
P4	Male	65	Primary School	married	Retired (driver)	Rural	11.5	86
P5	Female	52	Doctorate	married	Financial Consultant	City	27.9	80
P6	Male	29	Master’s Degree	Unmarried	Engineer	City	6.4	93
P7	Male	60	High School	married	Retired	Rural	34.9	75
P8	Female	58	Junior High School	divorced	attendant	Rural	73.4	55
P9	Male	49	High School	married	Self-employed	City	64.2	73
P10	Male	25	Master’s Degree	Unmarried	Student	Rural	25.5	72
P11	Male	42	Undergraduate	married	Mechanics	City	29.5	80
P12	Male	19	High School	Unmarried	Student	City	25.6	92
P13	Female	64	Primary School	married	Retired	City	65.6	78

**Table 4:** Characteristics of co-residents of patients with obstructive sleep apnea (*n* = 13).

Participant	Sex	Age	Educational level	Occupation	Relationship with the patient	Chronic diseases
C1	Female	58	Junior High School	self-employed	Spouse	Yes
C2	Female	36	High School	Sales	Spouse	No
C3	Female	50	High School	Civil Servants	mother	No
C4	Female	62	Primary School	Farmer	Spouse	Yes
C5	Male	52	Doctorate	Actuaries	Spouse	No
C6	Female	32	Master’s Degree	Medical personnel	mate	No
C7	Female	57	High School	Retired	Spouse	Yes
C8	Female	25	Undergraduate	Employee	daughter	No
C9	Female	49	Junior High School	self-employed	Spouse	No
C10	Male	24	Master’s Degree	Student	housemate	No
C11	Female	41	Undergraduate	Accountant	Spouse	No
C12	Male	43	Undergraduate	Civil Servant	father	Yes
C13	Male	62	High School	Retired	Spouse	Yes

A total of 318 initial entries were included in the study and 100 cards remained after excluding semantically repetitive entries. After the brainstorming method, 33 small group title cards, 15 medium group title cards and finally 7 large group title cards were used. The detailed K-J method analysis process is shown in Supplementary Figure 1. 7 themes and 18 sub-themes were finally revealed in this study. Table 5 shows the detailed information.

**Table 5:** The framework of themes and sub-themes.

Themes	Sub-themes
Theme 1: “The Shadow of Knowing Little “Cognitive deficit and response lag	Patient neglect of their disease
	Co-residents’ misperceptions of the disease
	Ignoring the impact of the disease and letting it run its course
Theme 2: “The Fog of Night and Day “Symptom Confusion and Difficulty of Awareness	Invisibility and diversity of symptoms
	Misattribution of symptoms
Theme 3: “Symbiotic Suffering “Physical and psychological well-being significantly affected	Impact on co-residents’ sleep quality
	Impact on co-residents’ psychological state
	Symptom clandestinity exacerbates distress
Theme 4: “The Hidden Costs “weakening social functions and causing economic losses	Safety hazards that come with the territory
	Reduced productivity at school
	Struggling with social interactions
Theme 5: “Delay in seeking medical care “Delay in seeking medical care, seeking information and guidance	Hospital visits driven by subjective feelings
	Hospital visits driven by external feedback
	Referrals due to co-morbidities
Theme 6: “Complex Choices “Questions about treatment options and prognosis	Confusion about treatment options
	Prognostic concerns
Theme 7: “Vacancies calling for attention “Insufficient popularization of social propaganda and insufficient professional facilities	Lack of social awareness
	Silos of technology

### 3.1 Theme 1: “The Shadow of Knowing Little”—cognitive deficit and response lag

#### 3.1.1 Patient neglect of their own disease

Patients often demonstrate a lack of awareness regarding sleep disorders, perceiving the symptoms as normal and even believing that they sleep well, despite being affected by the disease.

*All my friends snore too, and no one said it was a disease (P1).*

*I know very little about this disease, I do not even think it’s considered a disease, is not snoring very common? Everyone around me has this condition to a greater or lesser extent (P2).*

*When I was told that I was suffering from this sleep apnea condition, my first feeling was one of disbelief and I suspected that the doctor had made a mistake. I thought my sleep was fine and there was no problem at all (P6).*

#### 3.1.2 Misconceptions of disease among co-residents

The co-residents in question regarded the patient’s symptoms, such as snoring or insomnia, as commonplace and did not perceive the underlying health issues, which resulted in the patient’s condition being overlooked.

*We did not understand it either and did not see this snoring as a big problem. Isn’t it common for fatter people to snore? So it’s just been left alone with the kids (C12).*

*I think the patient is just getting older so he cannot sleep less, I do not think he is sick (C13).*

*He drives all the time and his routine is not regular, he snores in his sleep, pauses or whatever, I thought it was caused by him being too tired and did not think it was a disease (C4).*

#### 3.1.3 Minimizing the impact of the disease and allowing it to progress

Despite patients reporting sleep difficulties, they may perceive these issues as having minimal impact on their daily lives or as less pressing than other health concerns. This results in patients failing to devote sufficient attention to the disease, which may ultimately result in a worsening of the condition.

*I have been snoring for so many years, and I did not think it was a big problem, so I never went to the hospital (P7).*

*I have not been to the hospital because sleep apnea does not sound so serious, but high blood pressure is different. I think high blood pressure is a scary disease, so I do not think sleep apnea is a big concern compared to high blood pressure (P10).*

### 3.2 Theme 2: “The Fog of Night and Day”—symptom confusion and difficulty of awareness

#### 3.2.1 Insidiousness and diversity of symptoms

The symptoms of the disease are complex, encompassing both nocturnal sleep disturbances (e.g., snoring, apnea) and daytime neurological and emotional dysfunctions (e.g., drowsiness, headache, mood swings). This diversity and non-specificity of symptoms render early diagnosis challenging, and the disease’s resemblance to other conditions often leads to misdiagnosis, impeding patients’ ability to detect it. The insidious nature of the disease makes it challenging for patients and their families to identify its presence. It is not until the symptoms significantly impact their daily lives that they seek medical assistance. Furthermore, patients often fail to link nocturnal sleep disturbances with daytime symptoms such as reduced daytime energy and cognitive decline. They are frequently unaware of the correlation between these two sets of symptoms.

*In fact, all the symptoms of this disease occur during the daytime, and I have never associated these symptoms and discomfort that bother me with poor sleep at night, which also feels a bit strange, I guess (P5).*

*My child has stayed in grade 1 and is having a bit of trouble keeping up with the curriculum, so I wonder if this sleep disorder is the cause? (C12).*

*There is this condition of hypogonadism, and I wonder if it is caused by this disease? (P2).*

#### 3.2.2 Misattribution of symptoms

Patients may experience physical discomfort but frequently attribute their symptoms to alternative causes, such as stress-related insomnia, fatigue-induced headaches, or age-related memory loss. This tendency to self-diagnose can result in delayed medical attention, missing the optimal window for early intervention and treatment. Consequently, the condition may progress further, intensifying the patient’s suffering and burden on their co-residents.

*Because I have chronic pharyngitis, I thought my dry throat and chest tightness at night and that I could not breathe were caused by pharyngitis, and I did not think about the sleep aspect of it, I did not know it was caused by that (P4).*

*We all thought that our child was having problems with his emotions and had a tendency to be depressed, and we always looked in that direction, not thinking about the sleep aspect (C12).*

*I am old enough to think that this unresponsiveness and memory loss is common and do not connect these symptom with this disease (P13).*

### 3.3 Theme 3: “Symbiotic Suffering”—physical and psychological well-being significantly affected

#### 3.3.1 Impact on sleep quality of co-residents

The symptoms of nocturnal sleep disorders in patients can directly impact the quality of sleep of co-residents, resulting in a notable reduction in their overall sleep quality.

*I used to be in a house when I was younger and he used to snore like that, so loud I could not sleep, I had a nervous breakdown and almost got divorced (C4).*

*He gets up a lot at night and will stir, which affects my rest and my sleep is always forced to be interrupted (C6).*

*He snores exceptionally loudly, keeping several of us housemates awake, and there have been some minor disagreements over the matter (C10).*

#### 3.3.2 Impact on the psychological state of co-residents

The psychological distress and negative emotions experienced by co-residents as a result of the patient’s depression, fear and anxiety due to the disease can also have a detrimental impact on their well-being.

*Her discomfort either physically or mood-wise due to her illness affects me to some extent (C5).*

*My lover sometimes has apnea at night, he was snoring loudly and then suddenly stopped, it woke me up, I was so scared, I reached out to feel his nostrils and there was still air, in any case, he did not feel anything, but I was scared and could not sleep well the whole night (C2).*

*He has been suffering from apnea for many years, and recently it has worsened so much that I did not sleep at all, I just watched him, my heart clenched, and I did not dare to sleep (C9).*

#### 3.3.3 The insidious nature of the symptoms exacerbates the distress

The patient’s lack of awareness of their nocturnal symptoms results in co-residents being the first to identify them and subsequently experiencing the severe consequences. Those residing in the same space as the patient are forced to detect and cope with the patient’s symptoms, which has a detrimental impact on their physical and psychological wellbeing.

*He (the patient) is completely unaware of these conditions, and the disease is such that he himself does not feel anything, and I, who live with him, suffer both physically and psychologically (C9).*

*He does not know he’s (holding himself awake and pausing), I can hear a pause and a gasp when he’s asleep, it’s scary, it’s a disease that affects me a lot more than it does him (C1).*

### 3.4 Theme 4: “The Hidden Costs”—weakening social functions and causing economic losses

#### 3.4.1 The safety hazard that follows

The symptoms of the disease, such as drowsiness, render patients susceptible to accidents (e.g., traffic accidents) when driving, operating machinery or engaging in other activities that require concentration. This can result in financial losses and safety hazards.

*Two years ago, I was sleepy while driving and I almost fell asleep and collided with another minivan, I was fine but the car was smashed to pieces (P1).*

*I’m also sleepy when I ride my motorbike, sometimes I’m so sleepy that I fall asleep, I cannot even hold the handlebars, the front end of the bike is wobbly, (pointing to my bruised knee), and I fell down just the day before yesterday (P8).*

#### 3.4.2 Reduced learning and work efficiency

The disease causes daytime sleepiness, unresponsiveness, daytime depression, difficulty concentrating, and reduced learning and work efficiency.

*The symptoms of this disease (drowsiness) have seriously affected my daily life. I do not even dare to go to meetings now. When I was in a meeting before, I always fell asleep in the middle of the meeting and was named and criticized by the leader many times (P8).*

*I am sometimes particularly sleepy in class, I cannot keep my eyes open, and my grades have dropped severely (P12).*

#### 3.4.3 Struggling with social interactions

The disease may present with symptoms that impede patients’ ability to communicate effectively with others, thereby affecting their interpersonal interactions with co-residents, co-workers, clients, and fellow patients, and potentially leading to the deterioration of social relationships.

*He did this when he was young (snoring), then I got tired of him being too noisy and disturbing my sleep, and fought a lot because of this, and then separated (C1).*

*Emotional, moody, easily angry, in the course of work because of this reason, often fall out with colleagues (P2).*

*Due to my work, I often live with my colleagues, and because of my frequent snoring at night, my colleagues used to complain about me many times and ask for a change of room, and my colleague relationship was thus affected to some extent (P5).*

*I have got used to it for so many years. Whenever he snores in his sleep, my child still complains that her father is too noisy, which affects her homework, and then asks his father to close the bedroom door (P11).*

*When my dad was hospitalized before and I went to stay with him, the people in the same ward complained about me, saying that I snored too much in my sleep, and that I made sudden stops when I snored, which seriously affected their sleep, and then I was transferred to a single room (P1).*

### 3.5 Theme 5: “Delay in Seeking Medical Care”–delay in seeking medical care, seeking information and guidance

#### 3.5.1 Hospital visits driven by subjective feelings

Patients who are aware of the gravity of the condition due to the adverse effects it has on their wellbeing, such as a sense of suffocation, near-death experiences, severe insomnia, headaches and other symptoms, proactively seek information, guidance and medical assistance to gain clarity and address the underlying issue.

*In the first few years the symptoms (coughing, suffocating waking up) were not very obvious, did not take it seriously, but the day before yesterday I was half asleep and half waking up suffocating breath, a kind of choking sensation, stood up for a long time to relieve, my daughter-in-law and I were scared, and hurriedly came to the hospital for medical treatment (P7).*

*I used to have breath-holding at night, I usually got up and gasped for a couple of breaths, I did not think it was a big problem, but just two days ago I suddenly had a sense of suffocating on the verge of death, and that’s when I got scared and felt like I was going to die (P8).*

#### 3.5.2 Hospital visits facilitated by external feedback

Patients are prompted to seek medical attention ultimately by feedback from spouses, co-workers, or co-residents (expressing concern through words or actions).

*He had this snoring and apnea for about 5–6 years or so, and I could not stand it anymore when I kept fighting with him because of it, so I rushed to bring him to the hospital (C2).*

*I have friends who they were diagnosed with this disease, I learned some information about the disease from talking to them and gave me a lot of advice to come to the hospital (P11).*

#### 3.5.3 Referrals due to co-morbidities

Patients were referred for the presence of sleep disorders subsequent to examination for further information and guidance, as well as medical assistance due to the co-morbidities of other diseases, such as hypertension and diabetes mellitus.

*His hypertension has not been well controlled, and the doctor at the refractory hypertension clinic suggested that we look for any comorbid sleep disorders, and I’m actually a little confused about that - what do sleep disorders have to do with high blood pressure? (C2).*

*I’ve seen both Chinese and Western medicine, and I’ve been to 5–6 hospitals, but I cannot find the reason why his hypertension keeps worsening, is it related to sleep apnea? (C4).*

### 3.6 Theme 6: “Complex Choices”—questions about treatment options and prognosis

#### 3.6.1 Confusion over treatment options

Patients and their co-residents frequently inquire about the available treatment options and seek comprehensive information regarding the rationale, applicability, advantages, disadvantages, and costs associated with different treatment modalities. This is done in order to select the option that best aligns with their individual needs.

1 Patients and their co-residents would like to have an in-depth understanding of the principles of non-invasive positive pressure ventilation CPAP, how it is worn, adherence, and the cost of treatment.

*How much does a ventilator cost? What time of day do you need to wear the ventilator you bought for this treatment? (C4).*

*What is the best treatment plan for me? (P6).*

*I would like to know how this ventilator works. What is the difference between domestic and imported ventilator, movement? Or is the comfort level not quite the same? Can you recommend a better brand with a better user experience? (P5).*

*Do I need to maintain this ventilator regularly? (C6).*

2 Patients and their co-residents frequently inquire about the necessity of surgical intervention, the specifics of the procedure, the potential outcomes, and the efficacy of the proposed treatment. They seek assurance that the surgical approach will provide a definitive solution to their condition.

*Are there any side effects of the surgery to treat this condition? How does the surgery work? Do I have to have all these things cut out of my nose? That would be too much to bear (P2).*

*What are the other treatment options besides surgery, the safe and non-invasive but very effective kind? (C3).*

3 Patients and their co-residents require comprehensive guidance on daily living, encompassing the principles of side-lying, cessation of smoking and alcohol consumption, and structured programs for weight reduction, including dietary and exercise recommendations.

*I’ve been trying to lose weight but cannot get it off. Does this disease have anything to do with my heavier weight? Is there a good way to try to lose weight? (P8).*

*How can I treat this disease? What should I pay attention to in my daily life? (C10).*

#### 3.6.2 Disease prognosis concerns

Patients and their co-residents are concerned about the long-term effects and prognosis of the disease, including recovery status, complications, impact on quality of life and future life, and potential genetic risks.

*My youngest daughter is now 6 years old, my lover and I found that she also snores in her sleep, the sound is not very loud, and there is also the case of open mouth, are these symptoms of this disease? Is this a symptom of this disease? Will it affect the child’s growth and development? (C11).*

*What will happen to my body if I leave this disease untreated for a long time? (C9).*

*This sense of uncertainty about the disease makes me feel anxious, especially when I sleep at night, I often worry if I snore and suffocate to wake up, affecting my family’s rest (P3).*

*When I was told that I had this disease, there was also a bit of anxiety about how this disease would affect my health and future quality of life afterward (P5).*

### 3.7 Theme 7: “Vacancies Calling for Attention”—insufficient popularization of social propaganda and insufficient professional facilities

#### 3.7.1 Lack of social awareness

The absence of social publicity has resulted in a dearth of public comprehension of the disease, a superficial grasp of sleep disorders, and a deficiency in understanding the nature of the disease, its risks, and scientific treatment modalities. Consequently, patients frequently hold erroneous beliefs and delay seeking medical attention, which results in a postponement of treatment and the potential for missing the optimal treatment window.

*There was not enough publicity about the disease, I asked people around me and no one seemed to know that such a disease existed (P6).*

*People do not think of this disease as a disease, and there is a lack of awareness of this disease among the general public, I guess (C2).*

#### 3.7.2 Technology island

1 A plethora of sleep monitoring equipment is currently available on the market, yet the technological sophistication of these devices varies considerably. Moreover, the accuracy and reliability of some of these products are challenging to ascertain.

*I have done a sleep monitoring once before, and the facility is also rather rudimentary, it’s just a small box glued to the chest, called something portable, and finally gave a sleep monitoring report (P4).*

2 The proficiency of the operator varies considerably, with a notable absence of structured training. Errors in equipment operation and data interpretation diminish the reliability of sleep monitoring.

*The professor in the cardiology outpatient clinic said that the results of this sleep monitoring report we did before (prefecture-level city, second-class hospital) were inaccurate, and that there were a lot of contradictions in the data, so let us come to redo a sleep monitoring and verify it (P2).*

3 The absence of dedicated sleep monitoring units in prefecture-level hospitals hinders the provision of professional diagnosis and treatment to patients, thereby constraining the advancement and utilization of sleep monitoring technology.

*I wanted to go to my hometown to do sleep monitoring at first, but our local hospital did not have this sleep monitoring programs, so I came to your hospital to do the appropriate examination. You said that this sleep monitoring is still not popular, is it, not every hospital has it (P11).*

### 3.8 Analysis of interactions between themes

**Figure 1:**
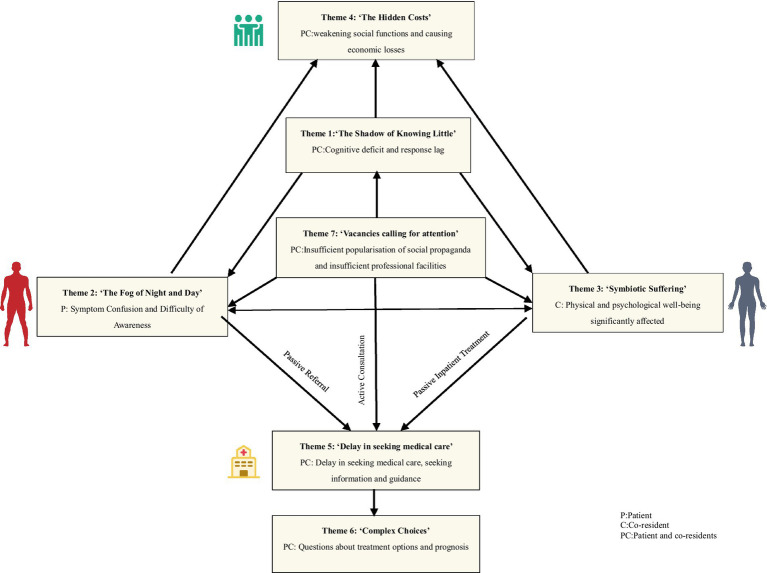
Chart of relationships between themes.

Analysis of interactions between themes: For further clarification, please refer to Figure 1 and Supplementary Figure 2, which illustrate the detailed relationship between the aforementioned concepts.

This study examines the multifaceted and interrelated challenges associated with managing the disease uncertainty experienced by individuals with OSA and their co-residents. Firstly, a lack of awareness and delayed response (Theme 1) are identified as key factors. Patients and their co-residents demonstrate an inadequate understanding of the symptoms, progression, and severity of OSA, resulting in delayed responses to symptoms occurring in themselves or in the patients. This directly exacerbates the difficulty of differentiating between symptoms (Theme 2) and contributes to physical and psychological impairment (Theme 3). The symptoms of OSA, such as snoring and daytime sleepiness, have a high degree of overlap with those of other diseases (Theme 2), increasing the difficulty of accurate identification and leading to delayed diagnosis (Theme 5). A delayed diagnosis and lack of disease recognition have a significant impact on the physical and psychological well-being of co-residents (Theme 3). These individuals experience physical distress, including poor sleep quality, daytime sleepiness, and reduced concentration, as well as negative emotions such as anxiety and depression. Additionally, they are subjected to stress and inconvenience due to the patient’s symptoms and disease. These effects further weaken the patient’s social functioning (Theme 4), resulting in socioeconomic losses, declining work capacity, and reduced quality of life. Furthermore, co-residents may be required to bear additional caregiving burdens and financial expenses. As a result, patients and co-residents frequently postpone seeking medical attention, opting instead to seek information and guidance (Theme 5). However, acquiring information is itself fraught with uncertainty about treatment efficacy, potential adverse effects, and long-term prognosis (Theme 6), further contributing to the perception of ambiguity surrounding the disease.

The root cause of these issues can be attributed to the inadequate dissemination of social information and the lack of accessible diagnostic and treatment resources (Theme 7), which has led to a lack of public awareness about OSA (Theme 1). This, in turn, has resulted in uncertainty among OSA patients and their co-residents, creating a complex and interacting vicious circle.

## 4 Discussion

In this study, 13 pairs of patients with obstructive sleep apnea and their co-residents were interviewed in depth using a binary conjoint interview method. The findings indicate that disease cognitive bias, discrepancies in symptom experience, challenges in medical decision-making, and inadequate social support collectively contribute to the disease uncertainty encountered by OSA patients and their co-residents. The following section will present a systematic elaboration of the manifestation, potential impacts and corresponding intervention strategies of OSA disease uncertainty at the individual, family and social levels. This will be achieved through an in-depth analysis that considers the existing literature.

### 4.1 Individual level: differential expression of perceptions and experiences

#### 4.1.1 Patient

Our findings align with prior studies highlighting the lack of awareness and cognitive biases among OSA patients (21, 22). This cognitive bias can be attributed to a lack of comprehension of the nature of the OSA disease and an underestimation of the gravity of the associated symptoms (23). Even when some patients are aware of sleep problems, they often have difficulty establishing a causal relationship between nighttime sleep abnormalities (such as snoring and apnea) and daytime fatigue, cognitive decline, and other symptoms (24). Patients may experience physical discomfort but frequently attribute their symptoms to alternative causes, such as stress-related insomnia, fatigue-induced headaches, or age-related memory loss, complications after the COVID-19 pandemic (25, 26). It is only when symptoms reach a certain severity that patients are compelled to seek medical attention. This reflects a passive, symptom-driven pattern of seeking medical care. To address this issue, it is imperative to enhance public awareness of the disease, underscoring its potential health hazards and the necessity of prompt intervention. Information should be disseminated via multiple channels, including community events, online platforms, and health education pamphlets, in order to reach as many people as possible.

#### 4.1.2 Co-resident

Our findings also support the critical role of co-residents in recognizing OSA symptoms early, as highlighted by Nufaiei et al. (27). However, our study extends these findings by exploring the dual burden faced by co-residents, who not only endure sleep disturbances but also experience heightened anxiety about the patient’s health. This dual burden aligns with existing evidence linking disease uncertainty to psychological distress in family member (28). In addition to the distress caused by their own sleep disorders, they must also consider the potential impact on the health and future of their patients (29). Furthermore, a significant number of co-residents assume the role of ‘disease discoverers’, thereby becoming a crucial driving force in the pursuit of information or in encouraging patients to seek medical intervention (30). Such external feedback and concerns frequently prove instrumental in prompting patients to seek medical assistance. Thus, OSA health education should target co-residents to improve symptom/danger recognition and family impact awareness. Recommended approaches include: educational materials, clinician consultations, health lectures for OSA knowledge enhancement, and psychological support to alleviate anxiety. Establishing peer support groups for experience-sharing is also effective.

### 4.2 Family level: disease interaction and relationship rift

#### 4.2.1 Fissures in family relations

The strain that OSA imposes on familial relationships has been well-documented (31, 32). Consistent with prior research, our study identifies sleep disturbances and mood swings as key drivers of conflict within families (33). The negative impact of OSA on family relationships is not limited to spousal relationships; it also affects patients’ relationships with their children, colleagues and other social relationships. This is evidenced by communication difficulties, interpersonal conflicts and even social isolation. Addressing this requires assisting families to accurately understand OSA, encouraging collective coping among co-residents, and strengthening familial support systems. Proven interventions include family therapy and couples’ communication training.

#### 4.2.2 Confusion about disease management

A paucity of information and understanding regarding the treatment and prognosis of OSA is evident among patients and their co-residents. Patients are keen to gain insight into the rationale, applicability, potential risks and long-term efficacy of different treatment modalities (e.g., CPAP, surgery) (34, 35). However, they frequently lack sufficient information and express concern about the long-term impact of the disease and potential complications (36). Furthermore, the issue of familial genetic risk frequently gives rise to feelings of anxiety among patients and their families (37). These uncertainties serve to exacerbate the anxiety and stress experienced by patients, which in turn affects the adherence and effectiveness of disease management. Physicians should enhance patient/family communication, develop individualized treatment plans, and provide ongoing support. Establishing mutual support platforms also facilitates experience-sharing and encouragement between patients and co-residents.

### 4.3 Societal level: the twin challenges of public perception and medical resources

#### 4.3.1 Lack of public awareness and lack of health education

One of the principal factors contributing to the uncertainty surrounding OSA is the lack of public awareness (38). The study participants reported a general lack of understanding of OSA, as well as insufficient knowledge of its symptoms, course, harms, and treatments. This lack of knowledge directly resulted in delays in seeking medical treatment and inappropriate disease management (39). This indicates that the general public’s knowledge of OSA remains superficial, with inadequate awareness of its gravity and potential consequences, impeding effective prevention and control of the disease (40). A nationwide OSA awareness program should be launched with tailored content for specific populations (e.g., elderly, parents). Multiple media channels (TV, radio, online, posters) can increase public attention (41). Community screening via portable devices and simple questionnaires is also essential for early high-risk detection.

#### 4.3.2 Imbalance in the allocation of medical resources and differences in treatment levels

Medical resource inequities exacerbate disease uncertainty for OSA patients and co-residents. Key issues include: inconsistent sleep monitoring equipment quality, insufficient technician training, and lacking specialized departments in prefecture-level/lower hospitals (42, 43). These directly compromise OSA diagnostic/treatment quality, delaying accurate diagnosis and effective management. Medical resources should be optimized, diagnostic and treatment levels improved, and sleep monitoring technology standardized and widely implemented. Professional training for medical personnel should be enhanced, and prefecture-level hospitals and above should establish specialized sleep medicine departments with appropriate equipment and staff to provide comprehensive services. Additionally, sleep disorder examination programs should be gradually introduced in county-level hospitals to reduce resource disparities.

## 5 Conclusion

This study, grounded in a theoretical model of illness uncertainty, explores the complex experiences of uncertainty faced by patients with obstructive sleep apnea and their co-residents. Key contributors include cognitive biases, symptom misattribution, decision-making difficulties, and inadequate support, which hinder timely interventions and effective disease management. To address these challenges, we propose a multilevel, evidence-based intervention framework.

At the individual level, educational campaigns should target misconceptions about OSA symptoms and their severity, highlighting the risks of delayed treatment and benefits of early intervention through platforms such as community health events, digital media, and tailored materials. At the family level, co-residents play a crucial role as ‘disease discoverers’ and facilitators of medical engagement. Supporting this role requires equipping them with knowledge to recognize OSA signs, offering psychological counseling to alleviate stress, and establishing family support groups to encourage collaborative disease management. At the societal level, addressing the unequal distribution of medical resources is critical. This includes establishing specialized sleep medicine departments in community hospitals, training healthcare providers, and initiating public health campaigns for OSA awareness and early screening.

These recommendations, derived from the study’s findings, aim to mitigate uncertainty, improve early diagnosis and treatment, and enhance the quality of life through a comprehensive, multidimensional strategy.

### 5.1 Limitations

This study has several limitations. First, it uses cross-sectional data, capturing perceptions of disease uncertainty among OSA patients and their co-residents at a single point in time, which limits the ability to observe changes over time. Future research should consider longitudinal designs to better understand the dynamics of disease uncertainty and the effectiveness of interventions. Second, the sample was limited to patients and co-residents from a single tertiary hospital in Shenyang, which may affect the generalizability of the findings due to geographic and medical care homogeneity. Additionally, qualitative research methods are inherently subjective, with findings potentially influenced by the researcher’s personal experience, cognitive ability, and analytical skills. Third, the study included unmarried patients (e.g., students) and divorced individuals, defining “co-residents” as housemates or the parents or children of divorced patients. These non-traditional family structures introduce variability in illness experiences and coping strategies. For instance, relationships between college students and housemates differ significantly from those within traditional family units, potentially leading to biased perceptions of OSA symptoms. Lastly, some single patients and their parents/children living in separate rooms may have unique dynamics that constrain their narratives, creating discrepancies between perceived and actual disease uncertainty. Future studies should carefully evaluate such special samples, ensuring that data analysis accounts for sample heterogeneity to maintain reliability.

## Data Availability

The original contributions presented in the study are included in the article/Supplementary material, further inquiries can be directed to the corresponding author.
