# Supplementary figures and images for: Social and environmental determinants of disease uncertainty in obstructive sleep apnea: a dyadic qualitative study on patients and co-residents
Source: Front Neurol. 2025 Aug 12;16:1582173. doi: 10.3389/fneur.2025.1582173 (PMC12378041; doi:10.3389/fneur.2025.1582173)

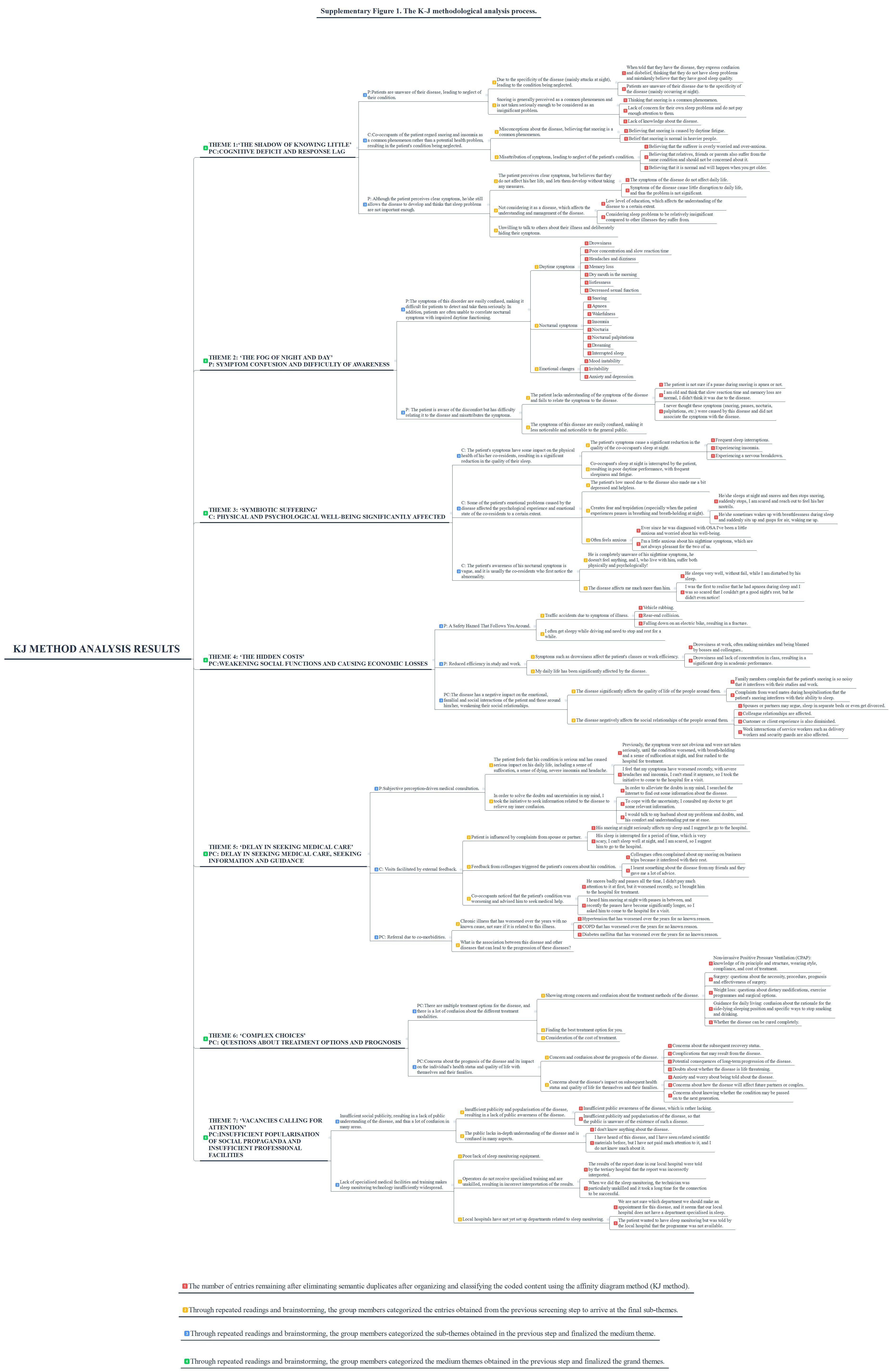

Supplement: Supplementary file 1 [file Image_1.jpeg]

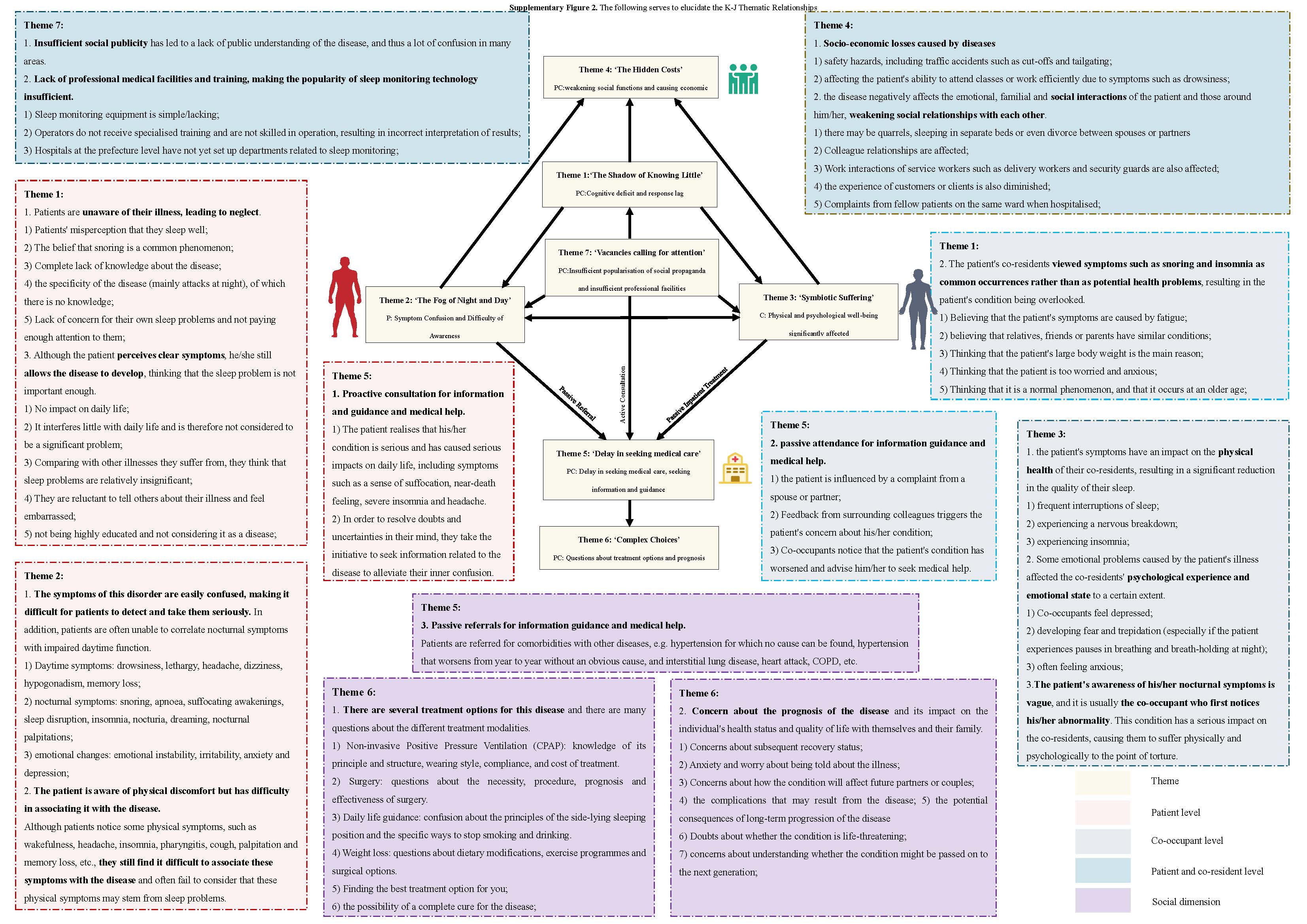

Supplement: Supplementary file 2 [file Image_2.jpeg]
